# Salivary cortisol levels and anxiety in melanoma patients undergoing sentinel lymph node excision under local anesthesia versus general anesthesia: a prospective study

**DOI:** 10.1186/s12957-020-01823-w

**Published:** 2020-03-10

**Authors:** Philipp Jansen, Ingo Stoffels, Anne-Christine Müseler, Maximilian Petri, Titus J. Brinker, Manfred Schedlowski, Dirk Schadendorf, Harald Engler, Joachim Klode

**Affiliations:** 1 Department of Dermatology, Venereology and Allergology, University Hospital Essen, University of Duisburg-Essen, Hufelandstraße 55, 45147 Essen, Germany; 2 West German Cancer Center, University Duisburg-Essen, Essen, Germany; 3 German Cancer Consortium, Heidelberg, Germany; 4 Department of Dermatology, University Hospital Heidelberg, Heidelberg, Germany; 5 National Center for Tumor Diseases, German Cancer Research Center, Heidelberg, Germany; 6 Institute of Medical Psychology and Behavioral Immunobiology, University Hospital Essen, University of Duisburg-Essen, Essen, Germany

**Keywords:** Perioperative salivary cortisol level, Stress, Anxiety, Anesthesia, Melanoma

## Abstract

**Background:**

Sentinel lymph node excision (SLNE) can be performed in tumescent local anesthesia (TLA) or general anesthesia (GA). Perioperative cortisol level changes and anxiety are common in surgical interventions and might be influenced by the type of anesthesia. In this study, we intended to determine whether the type of anesthesia impacts the patients’ perioperative levels of salivary cortisol (primary outcome) and the feeling of anxiety evaluated by psychological questionnaires (secondary outcome).

**Methods:**

All melanoma patients of age undergoing SLNE at the University Hospital Essen, Germany, could be included in the study. Exclusion criteria were patients’ intake of glucocorticoids or psychotropic medication during the former 6 months, pregnancy, age under 18 years, and BMI ≥ 30 as salivary cortisol levels were reported to be significantly impacted by obesity and might confound results.

**Results:**

In total, 111 melanoma patients undergoing SLNE were included in our prospective study between May 2011 and April 2017 and could choose between TLA or GA. Salivary cortisol levels were measured three times intraoperatively, twice on the third and second preoperative day and twice on the second postoperative day. To assess anxiety, patients completed questionnaires (Hospital Anxiety and Depression Scale (HADS), State-Trait Anxiety Inventory (STAI)) perioperatively. Patients of both groups exhibited comparable baseline levels of cortisol and perioperative anxiety levels. Independent of the type of anesthesia, all patients showed significantly increasing salivary cortisol level from baseline to 30 min before surgery (T3) (TLA: *t* = 5.07, *p* < 0.001; GA: *t* = 3.09, *p* = 0.006). Post hoc independent *t* tests showed that the TLA group exhibited significantly higher cortisol concentrations at the beginning of surgery (T4; *t* = 3.29, *p* = 0.002) as well as 20 min after incision (T5; *t* = 277, *p* = 0.008) compared to the GA group.

**Conclusions:**

The type of anesthesia chosen for SLNE surgery significantly affects intraoperative cortisol levels in melanoma patients. Further studies are mandatory to evaluate the relevance of endogenous perioperative cortisol levels on the postoperative clinical course.

**Trial registration:**

German Clinical Trials Register DRKS00003076, registered 1 May 2011

## Background

The excision of the first draining lymph node (sentinel lymph node; SLN) is a routinely performed surgical intervention in melanoma patients [1–3]. It can be done using tumescent local anesthesia (TLA) or general anesthesia (GA) [4–7], mainly depending on the patients’ preference. The relevance of patients’ perioperative anxiety and stress has been an area of great interest for the past decades. Elevated levels of stress could be shown to impair the inflammatory response and led to slower wound recovery [8]. In patients undergoing cholecystectomy, induced relaxation led to lower corticosteroid levels and less surgical wound erythema [9]. Accordingly, anxiety measured by cortisol levels significantly altered the number of lymphocytes when non-malignant skin alterations were excised [10] and negatively correlated with the speed of wound healing after punch biopsy of the skin [11]. Reducing preoperative stress and anxiety may improve wound healing, patients’ adherence to the doctors’ advice, and shorten hospital stay [12]. To our knowledge, the impact of the type of anesthesia (TLA vs. GA) on perioperative anxiety and cortisol levels has not been investigated in melanoma patients undergoing sentinel lymph node excision before and might further contribute to addressing the patients’ concerns and postoperative complications. The aim of our study was to determine the impact of the type of anesthesia on cortisol levels as a surrogate of stress during SLN excision. The perioperative secretion levels of cortisol were correlated with patients’ perioperative feeling of anxiety.

## Methods

### Patients and sample size calculation

All patients of age undergoing a sentinel lymph node excision at the Department of Dermatology, University Hospital Essen, Germany, due to a melanoma of stages I and II according to AJCC 2009 (American Joint Committee on Cancer) could be included in our prospective study. Exclusion criteria included intake of glucocorticoids or psychotropic medication during the former 6 months, pregnancy, age under 18 years, and BMI ≥ 30 as salivary cortisol levels were reported to be significantly impacted by obesity and might confound results [13]. We calculated with two groups of the same size to determine the sample size by means of the independent *t* test: group 1 = group 2, standard deviation 1 = standard deviation 2. Group 1 is the number of melanoma patients undergoing sentinel lymph node excision in tumescent local anesthesia (TLA). Group 2 is the number of melanoma patients undergoing sentinel lymph node excision in general anesthesia (GA). The differences of mean values between the two groups were denoted as *μ*_1_ and *μ*_2_. The power was expected at 80% (*Z*(0.8) = 0.8416) and the level of significance was set at 2.5% (*Z*(0.975) = 1.96). The expected difference in mean value was 5 nmol/l of salivary cortisol level and a standard deviation (*δ*) of 10 nmol/l. In this calculation, *α* is the level of significance and *Z*_1−*α*_ is the quantile of standard deviation [14].

$$ n\approx {\left[\frac{2\left({Z}_{\mathrm{Power}}+{Z}_{1-\upalpha}\right)}{\raisebox{1ex}{$2\left({\mu}_1-{\mu}_2\right)$}\!\left/ \!\raisebox{-1ex}{$\updelta $}\right.}\right]}^2 $$

We calculated that the total number of patients included in our prospective study should be 126. As patients should be equally distributed into two groups, we intended to include 63 patients in each of our two groups (TLA and GA).

### Study design

**Fig. 1:**
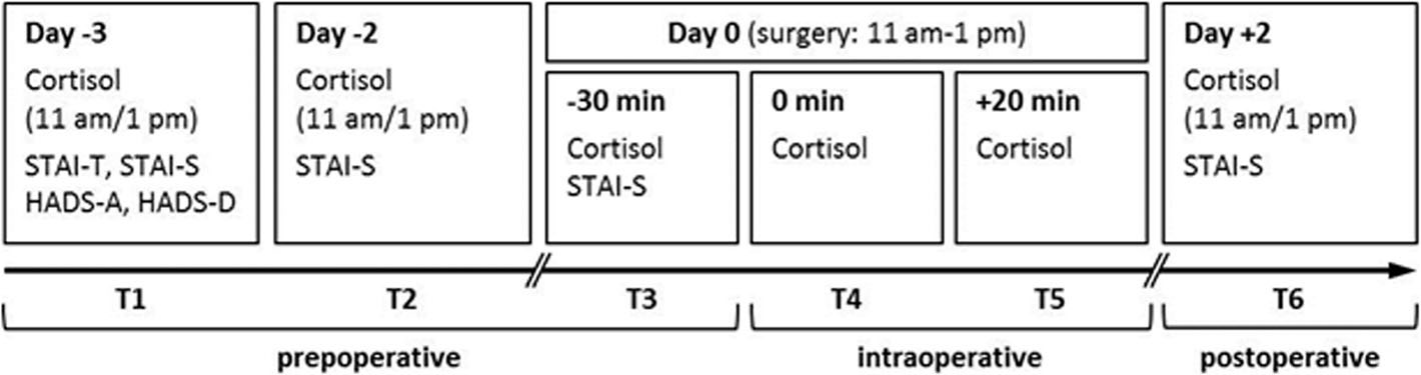
Study design. Melanoma patients scheduled for SLNE were allocated into two groups: those receiving TLA and those receiving GA during surgery. Group assignment was based on patient preference. Saliva for cortisol measurements was collected on preoperative days (T1, T2), on the day of surgery (T3–T5), and 2 days after surgery (T6). In addition, patients had to complete psychological questionnaires at indicated time points. STAI, State-Trait Anxiety Inventory; HADS, Hospital Anxiety Depression Scale

Patients scheduled for sentinel lymph node excision (SLNE) at the Department of Dermatology, University Hospital Essen, Germany, were offered to participate in the study. The participating patients were allocated into two groups according to personal preference: one group operated in tumescent local anesthesia (TLA) and one group operated in general anesthesia (GA). The surgical intervention was performed between 11 am and 1 pm to minimize the confounding effects of diurnal variation in cortisol secretion. Saliva for cortisol measurements was collected twice (11 am and 1 pm) on preoperative days 3 (T1) and 2 (T2), at three occasions on the day of surgery (30 min before surgery [T3], at skin incision [T4], and 20 min [T5] after beginning of surgery, respectively), and twice (11 am and 1 pm) on postoperative day 2 (T6). In the GA group, saliva collection during surgery (T4 and T5) was performed by the anesthesiologist (Fig. 1). In addition, all patients had to complete psychological questionnaires at indicated time points (see the “Psychological questionnaires” section for details).

### Anesthesia

SLNE was performed either under TLA or GA. TLA was originally described by Lillis for liposuction surgery [15]. We used a 0.1% TLA solution with the following composition: 450 mL physiological sodium chloride solution 0.9% (B. Braun Melsungen AG, Melsungen, Germany), 50 mL lidocaine 1% (Xylocain® 1% AstraZeneca GmbH, Wedel, Germany) and 0.5 mg epinephrine (Sanofi Aventis, Frankfurt, Germany). The amount of lidocaine (1%) used in TLA was always less than the recommended safe dose of 35 mg/kg body weight. The GA was induced by remifentanil (0.5 μg/kg) and maintained with a target-control infusion of propofol (effector site concentration, 4 μg/ml). In the vast majority of cases, airway management was performed by a laryngeal mask airway. The decision between laryngeal mask airway (LMA) or tracheal intubation was made by the anesthesiologist dependent on the intraoperative need for inversion of the patient. Cis-atracurium (0.15 μg/kg) was used as a muscle relaxation in case of endotracheal intubation. Pressure-controlled tidal ventilation was set at 8 ml/kg during bilateral ventilation with a peak airway pressure that did not exceed 25–30 mmHg. Intraoperative high inspired oxygen fraction was adjusted at the minimum fraction necessary to keep the oxygen saturation above 95%. All patients (TLA and GA) orally received midazolam (7.5 mg) ± 1 h to incision. No non-pharmacological methods such as music via headphones or hypnosis were offered as additional relaxation techniques. Factors known to influence anxiety and preoperative stress such as friendly ambience, calm, and open attitude were kept comparable across patients.

### Sentinel lymph node excision (SLNE)

The surgical technique was identical in both groups, and operating physicians had an experience of more than 100 SLNEs per year. The procedure has been described previously [16].

### Salivary cortisol analysis

Saliva samples were obtained with commercially available collection devices (Salivette Cortisol, Sarstedt, Nümbrecht, Germany). Saliva was recovered by centrifugation (1000×*g*, 2 min, 4 °C) and was stored at − 20 °C until assayed. Salivary cortisol concentrations were measured using an enzyme-linked immunosorbent assay (Cortisol ELISA, IBL International, Hamburg, Germany) according to the manufacturer’s instructions. Cross-reactivity of the anti-cortisol antibody with other relevant steroids was 7.0% (11-deoxycortisol), 4.2% (cortisone), 1.4% (corticosterone), 0.35% (progesterone), and < 0.01% (testosterone, estrone, estradiol, estriol). Intra- and interassay variances were 4.8% and 5.9%, respectively. For comparison of cortisol data from pre- and postoperative days with the day of surgery, salivary cortisol levels of the two collection times (11 am and 1 pm) on preoperative day 3 (T1), on preoperative day 2 (T2), and on postoperative day 2 (T6) were averaged. By measuring the cortisol levels 2 days after surgery, we intended to circumvent that elevated levels of cortisol induced by immediate postoperative pain might have confounded evaluation of anxiety.

### Psychological questionnaires

The Hospital Anxiety and Depression Scale (HADS) was used to screen for ongoing affective disturbances and to quantify subclinical symptoms of depression and anxiety [17]. The HADS is a validated 14-item questionnaire that can be divided into two subscales for anxiety (HADS-A) and depression (HADS-D). Both subscales contain seven Likert-scaled items, with sum scores ranging from 0 to 21. Higher sum scores indicate higher levels of anxiety and depression, respectively (scores < 7 indicate normal range, scores from 8 to 10 reflect mild alterations, scores ≥ 11 indicate clinically relevant symptoms). Trait and state anxiety were assessed using the validated State-Trait Anxiety Inventory (state version, STAI-S; trait version, STAI-T) [18], with higher sum scores indicating higher levels of state/trait anxiety. Sum scores > 40 are considered to reflect clinical symptoms of anxiety [19]. The STAI-S was completed by all patients on preoperative days 3 (T1) and 2 (T2), 30 min before surgery (T3), and on postoperative day 2 (T6).

### Statistical analysis

The primary outcome measure was the salivary cortisol level as a surrogate marker for stress in melanoma patients undergoing sentinel lymph node excision either under tumescent local anesthesia or under general anesthesia. The secondary outcomes were results of psychological questionnaires (HADS-A, HADS-D, STAI-S, and STAI-T) perioperatively edited by the participating melanoma patients. Data analysis was performed using SPSS 20.0 (SPSS Inc., Chicago, IL, USA). Normality of data distribution was examined using the Shapiro-Wilk test. Sociodemographic and clinical data were analyzed by chi-square tests (categorical data) and *t* tests (continuous data), respectively. Salivary cortisol and state anxiety data were analyzed by repeated-measures analysis of variance (ANOVA) with “group” (TLA vs. GA) as the between-subject factor and “time” as the within-subject factor. Independent or paired *t* tests were used for post hoc comparisons. The level of significance was set at *p* < 0.05. All data are presented as mean and standard error of the mean (SEM).

## Results

### Patient characteristics

**Fig. 2:**
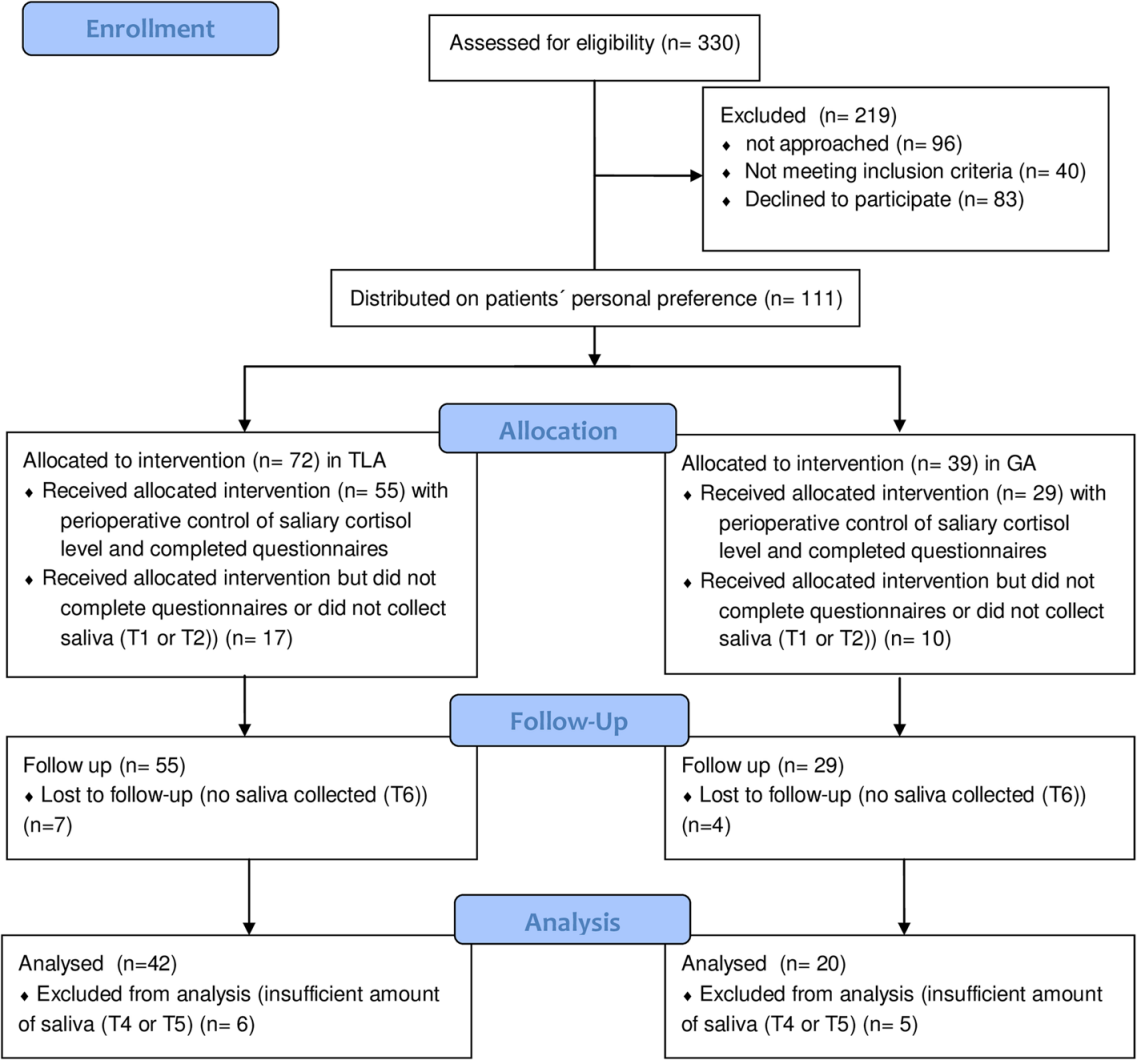
Selection of patients

Between May 2011 and April 2017, 330 patients with malignant melanoma in AJCC (American Joint Committee on Cancer) stages I and II were assessed for eligibility. Still, 219 patients could not be included as they did not meet inclusion criteria (*n* = 40), declined to participate (*n* = 83), or could not be approached at least 4 days prior to surgical intervention to give saliva (*n* = 96). From 111 patients included in our prospective trial, 72 patients preferred to undergo SLNE in TLA and 39 patients preferred to undergo SLNE in GA. Discharged on the second postoperative day in the morning, 30 patients from the TLA group and 19 patients from the GA group were lost to follow-up, forgot to collect saliva at 11:00 am/1:00 pm, or lost their saliva collectors. Thus, we could analyze saliva cortisol levels of 42 patients operated in TLA and 20 patients operated in GA (Fig. 2). The included patients (mean age = 54.4 ± 2.6 years, range 18–82 years) undergoing SLNE in TLA (*n* = 42) and GA (*n* = 20) did not significantly differ in age, gender, clinical characteristics (tumor depth, localization of the primary tumor, ulceration rate, and localization and number of SLNs) and psychological variables at baseline (Table 1). At the termination of the study, the patients were followed-up for a mean time of 45 months.

**Table 1:** Sociodemographic and clinical characteristics

Variable	TLA (*n* = 42)	GA (*n* = 20)	*P* value^b^
Age (years)^a^	56.7 ± 2.7	52.1 ± 2.5	0.28
Gender (female)	18 (42.9%)	8 (40%)	0.83
Gender (male)	24 (57.1%)	12 (60%)	
Tumor depth (mm)^a^	2.16 ± 0.34	2.70 ± 0.86	0.48
Primary localization			0.24
Head/neck	2 (4.8%)	3 (15.0%)	
Trunk	17 (40.5%)	11 (55.0%)	
Upper extremity	10 (23.8%)	3 (15.0%)	
Lower extremity	13 (31%)	3 (15%)	
Ulceration (present)	8 (19%)	6 (30%)	0.52
Ulceration (absent)	34 (81%)	14 (70%)	
Localization of SLNs			0.47
Cervical	4 (9.5%)	3 (15%)	
Axillary	26 (61.9%)	14 (70%)	
Inguinal	12 (28.6%)	3 (15%)	
SLNs per patient^a^	2.17 ± 0.19	2.25 ± 0.42	0.84
Baseline cortisol levels (T1) (nmol/l)	12.7 ± 0.9	14.7 ± 2.2	0.33
HADS score^a^			
HADS-A (anxiety)	7.5 ± 0.7	7.6 ± 1.1	0.95
HADS-D (depression)	4.6 ± 0.6	4.3 ± 0.8	0.76
STAI score^a^			
STAI-S (state)	42.4 ± 2.2	42.2 ± 3.1	0.96
STAI-T (trait)	37.5 ± 1.6	36.7 ± 2.1	0.77

^a^Data are presented as mean ± SEM

^b^Group differences were analyzed using chi-square tests (categorical data) and *t* tests (continuous data)

*LA* local anesthesia, *GA* general anesthesia, *HADS* Hospital Anxiety Depression Scale, *STAI* State-Trait Anxiety Inventory

### Salivary cortisol levels

**Fig. 3:**
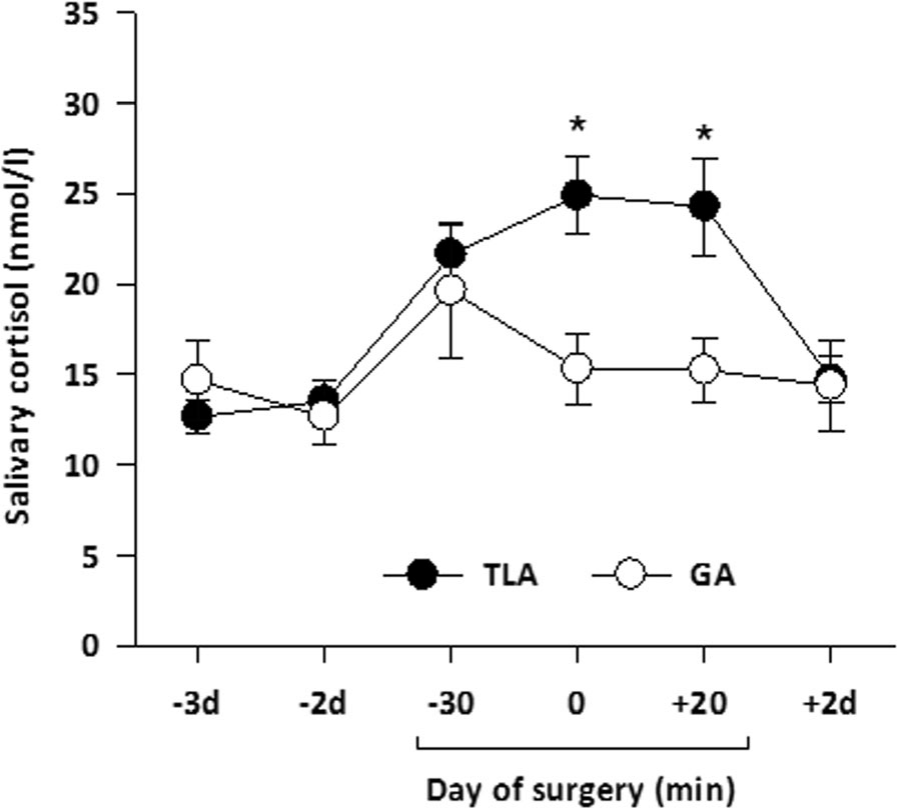
Salivary cortisol concentrations during preoperative, intraoperative, and postoperative phases in patients undergoing SLNE under local anesthesia (TLA; *n* = 42) or under general anesthesia (GA; *n* = 20). Means ± SEM are shown. *p < 0.05 (Bonferroni-corrected)

Initial values (T1) for salivary cortisol concentration did not significantly differ between TLA and GA groups (12.7 ± 0.9 vs. 14.7 ± 2.2 nmol/l; independent *t* test: *t* = 0.98, *p* = 0.331). However, repeated measure ANOVA on cortisol data revealed significant effects of time (*F* = 8.90, *p* < 0.001), group (*F* = 4.59, *p* = 0.045), and time × group interaction (*F* = 3.37, *p* = 0.019). Post hoc paired *t* tests showed that salivary cortisol levels significantly increased from baseline to 30 min before surgery (T3) in both groups (TLA: 21.6 ± 1.7 nmol/l, *t* = 5.07, *p* < 0.001; GA: 19.6 ± 3.7 nmol/l, *t* = 3.09, *p* = 0.006). Moreover, the type of anesthesia had a marked impact on cortisol secretion during surgery. At the beginning of surgery, salivary cortisol further increased in the TLA group, while it dropped in the GA group. Post hoc independent *t* tests showed that the TLA group exhibited significantly higher cortisol concentrations at the beginning of surgery (T4; 24.9 ± 2.2 nmol/l (TLA) vs. 15.3 ± 1.9 nmol/l (GA), *t* = 3.29, *p* = 0.002) as well as 20 min after incision (T5; 24.3 ± 2.7 nmol/l (TLA) vs. 15.2 ± 1.8 nmol/l (GA), *t* = 2.77, *p* = 0.008) compared to the GA group. The cortisol level of the GA group resembled cortisol levels measured on preoperative days. Two days after surgery, cortisol levels in both groups (TLA, 14.7 ± 1.3 nmol/l; GA, 14.4 ± 2.6 nmol/l) were back to levels measured on preoperative days (Fig. 3).

### State anxiety levels

To assess the anxiety and stress of patients, questionnaires were answered by all patients. State anxiety levels did not significantly differ during pre- and postoperative periods between patients in TLA and GA groups (Table 2). However, repeated measure ANOVA revealed a significant effect of time (*F* = 25.65, *p* < 0.001). Planned post hoc paired *t* tests showed a significant increase in state anxiety from preoperative day 3 (T1) to 30 min before surgery (T3) in both the TLA (*t* = 3.74, *p* = 0.001) and the GA (*t* = 3.64, *p* = 0.002) groups. Postoperative state anxiety (T6) was in both groups significantly lower compared to baseline (paired *t* tests, TLA: *t* = 3.77, *p* = 0.001; GA: *t* = 2.24, *p* = 0.038).

**Table 2:** State anxiety levels

STAI-S score	LA (*n* = 42)	GA (*n* = 20)	*P* value^a^
Preoperative, 3 days (T1)	42.4 ± 2.2	42.2 ± 3.1	0.96
Preoperative, 2 days (T2)	44.4 ± 2.1	45.7 ± 3.5	0.74
Preoperative, 30 min (T3)	48.2 ± 2.1	48.5 ± 3.3	0.93
Postoperative, 2 days (T6)	35.5 ± 1.5	36.9 ± 2.4	0.61

Data are presented as mean ± SEM

^a^Data were analyzed by repeated measure ANOVA (time effect: *F* = 25.65, *p* < 0.001; group effect: *F* = 25.65, *p* = 0.82; time × group interaction: *F* = 0.15, *p* = 0.93) followed by post hoc independent *t* tests

*LA* local anesthesia, *GA* general anesthesia, *STAI-S* State-Trait Anxiety Inventory situation

### Intra- and postoperative clinical course

The length of surgery measured from incision till full closure of the wound took between 60 and 120 min mainly depending on the SLN basins operated. We could neither determine a significant time difference in length of surgery nor a significant impact of surgery time on cortisol level between the two groups. All preoperative preparations (applicating TLA and initiating GA) could be performed within the 30-min slot between T3 and T4 (Fig. 1). Both in the TLA and in the GA group about 45% of patients asked for postoperative analgesics (TLA 20/42 vs GA 9/20). On average, patients of both groups could be discharged from the hospital 2 days after surgery. There was no statistically significant difference in the duration of hospital stay between the two groups. During postoperative hospital stay and follow-up examinations, we could determine complications like wound infections and lymph drainage disorders in three patients of the GA group (3/42) and one patient of the TLA group (1/20).

## Discussion

In our study, we correlated perioperative endogenous cortisol level and anxiety in melanoma patients undergoing the same surgical intervention in either local anesthesia (TLA) or general anesthesia (GA). Patients undergoing sentinel lymph node excision (SLNE) in TLA and GA did not significantly differ in baseline salivary cortisol values, and independent of the type of anesthesia chosen, both groups displayed a similar increase in salivary cortisol directly before surgery. The intraoperative cortisol levels of the GA group in our study were similar to the preoperative levels of both groups. This observation can be underlined by patients undergoing thyroid surgery in GA. Their cortisol level remained within the preoperative range as well [20]. However, patients of our study operated in GA displayed significantly lower cortisol levels compared to patients in TLA. In the TLA group from our study, we determined a twofold increase in salivary cortisol level compared to baseline (Fig. 3). Our results are in line with previously reported perioperative cortisol response during carotid endarterectomy (CEA) performed in LA and GA [21]. Accordingly, a study observing perioperative salivary cortisol levels in response to varying levels of sedation found that 25% of patients had a fourfold increase in salivary cortisol levels and mean cortisol values increased more than threefold from baseline if surgical intervention was performed in LA [13]. In contrast to these findings, Hill and Walker investigated the salivary cortisol levels in patients undergoing third molar removal under LA and GA. Their results showed that patients treated in LA showed lower levels of stress response than those having treatment in GA [22]. To evaluate the influence of the type of anesthesia on cortisol level, the severity of surgical intervention must possibly be regarded as well. In a study from 2017, it was shown that peak cortisol levels positively correlated with the severity of surgical intervention [23]. Severity might additionally prolong the postoperative duration of elevated cortisol levels. We determined cortisol levels comparable to preoperative levels on the second postoperative day. In contrast, interventions like coronary artery bypass grafting showed more persistent elevations of cortisol at least for 2 postoperative days [24]. As the severity of surgical intervention was the same in our two groups, medications used to induce GA might impact cortisol levels. Fentanyl is commonly used for GA. It is known to abolish cortisol secretion in cardiac surgery [25]. Accordingly, propofol in higher doses averts secretion of cortisol during surgery as well [26]. From these observations follow that medically induced suppression of corticosteroid secretion might oppose physiological rise in cortisol level. This prevention of rise in cortisol levels of the GA group might even be of medical benefit. Antibody-mediated blockage of glucocorticoid receptor in animal trials led to equally fast wound healing in stressed mice compared to control group [27]. During the follow-up period of our patients for more than 45 months in mean, we could not see a difference between the two groups with regard to postoperative complications like wound infections. Beside the severity of surgery and medications used, anxiety may have an additional effect on intraoperative cortisol levels. To further correlate the cortisol levels and address the feeling of anxiety, we asked all patients to fill questionnaires perioperatively. In comparison to the general population, state anxiety levels in our patient cohorts were markedly elevated on preoperative days and increased even further on the day of surgery [28]. This is in line with previous interrogations of patients with different oncological or hematological types of cancer awaiting surgery [29]. Elevated state anxiety level and history of cancer are known to favor preoperative anxiety [30]. The results of the questionnaires correlated with the preoperative increase in cortisol levels of both groups. Perioperative trait/state anxiety levels assessed by questionnaires were highly comparable between TLA and GA groups. We could not conclude that patients with higher preoperative anxiety preferred either form of anesthesia. Anxiety might worsen patients’ perception of pain and increase requirements for postoperative analgesia [31–33]. Choi et al. examined that elevated cortisol levels negatively correlated with pain threshold when participants suffered from pain induced by electrical stimulation [34]. Hence, elevated salivary cortisol levels and anxiety might be regarded as an indicator for patients’ demand for analgesics. In our study, both cortisol levels and anxiety were comparable between the two groups on the second postoperative day. We could not determine that the intraoperative difference in cortisol had an effect on postoperative demand for analgesia. State anxiety levels of both groups postoperatively dropped below levels prior to surgery indicating a possible sign of relief. Our results might underline that patients undergoing surgery predominantly fear postoperative pain and postoperative nausea as previously described by Mavridou et al. [35]. Those concerns could be addressed as part of preoperative management. Our study is limited by the small number of patients. Although our results are statistically relevant, the clinical relevance is possibly limited. As a fourth of all patients denied to participate (83/330), it cannot be excluded that patients with specific characteristic traits (e.g., elevated levels of fear) tended to deny participation in our study leading to selection bias and limited applicability of our data. Thus, the limited number of patients included in our study might have concealed a possible preference for any type of anesthesia based on stress or fear and a possible impact of perioperative cortisol levels on the patients’ clinical course (e.g., wound healing).

## Conclusion

Based on our findings, melanoma patients undergoing sentinel lymph node excision under tumescent local anesthesia (TLA) show significantly elevated cortisol concentrations at the beginning and 20 min after excision compared to those under general anesthesia (GA). Further studies are mandatory to evaluate the relevance of endogenous perioperative cortisol levels on the postoperative clinical course.

## Data Availability

The datasets used and/or analyzed during the current study are available from the corresponding author on reasonable request.

## Abbreviations

AJCC: American Joint Committee on Cancer
BMI: Body mass index
CEA: Carotid endarterectomy
GA: General anesthesia
HADS: Hospital Anxiety and Depression Scale
HADS-A: Subscale of HADS questionnaire to assess anxiety
HADS-D: Subscale of HADS questionnaire to assess depression
SLNE: Sentinel lymph node excision
STAI: State-Trait Anxiety Inventory
STAI-S: State version of STAI
STAI-T: Trait version of STAI
TLA: Tumescent local anesthesia
